# Supplementary material for: The Italian Validation of the Zimbardo Time Perspective Inventory and Its Comparison with Three Time Perspective Inventories
Source: Int J Environ Res Public Health. 2023 Jan 31;20(3):2590. doi: 10.3390/ijerph20032590 (PMC9915386; doi:10.3390/ijerph20032590)
Supplement: Supplementary file 1 [file ijerph-20-02590-s001.zip › ijerph-2109600-Supplementary Table S1.pdf]

Zimbardo Time Perspective Inventory short version with high frequent items (ZTPI-16)

List of items of the Italian short version of the ZTPI, the ZTPI-16 composed by high frequent items of previous short versions. We report the numbers of the corresponding item of the ZTPI-56. In parenthesis, the subscale which the single item is referred to, as well as an English translation of the Italian item are reported. In the following part of this Supplementary Material, we report the Italian instructions for the administration.

La preghiamo di leggere le affermazioni che seguono e di rispondere, il più sinceramente possibile alla domanda: **"In che misura questa affermazione è vera o rappresentativa della mia persona?"**. Le chiediamo di rispondere a *tutte* le domande. Contrassegni il quadratino appropriato, ricordandosi che la scala indica:

1= per nulla vero (completamente falso

2= non del tutto vero (abbastanza falso)

3=né vero, né falso

4= abbastanza vero

5= del tutto vero

ZTPI-56	ZTPI-16		1	2	3	4	5
2	1	Immagini, suoni e odori caratteristici della mia infanzia spesso ridestano in me una folla di bellissimi ricordi (PP) [*Family childhood, sights, sounds, smalls often bring back a flood of wonderful memories*]					
7	2	Mi piace pensare al mio passato (PP) [I*t gives me pleasure to think about my past*]					
13	3	Rispettare le scadenze di domani e non restare indietro col lavoro è più importante del divertimento di stasera (F) [*Meeting tomorrow's deadlines and doing other necessary work comes before tonight's play*]					
16	4	Le brutte esperienze del passato continuano a tornarmi in mente (PN) [*Painful past experiences keep being replayed in the mind*]					
20	5	I ricordi felici dei tempi belli mi vengono in mente con facilità (PP) [*Happy memories of good times spring readily to mind*]					
21	6	Adempio puntualmente ai miei doveri verso gli amici e le autorità (F) [*I meet my obligations to friends and authorities on time*]					
26	7	È importante che nella mia vita vi siano momenti eccitanti (PH) [*It is important to put excitement in my life*]					
31	8	Fare cose rischiose è un buon modo per non annoiarsi (PH) [*Taking risks keeps my life from becoming boring*]					
34	9	Vi sono immagini sgradevoli della mia infanzia che ho difficoltà a dimenticare (PN) [I*t's hard for me to forget unpleasant images of my youth*]					
37	10	È praticamente impossibile fare programmi per il futuro, perché le cose cambiano troppo (PF) [*You can't really plan for the future because the things change so much*]					
38	11	Il corso della mia vita è governato da forze che non sono in grado di controllare (PF) [*My life path is controlled by forces I cannot influence*]					
39	12	Non ha senso che mi preoccupi del futuro, perché non c'è nulla che possa fare al riguardo (PF) [*It doesn't make sense to worry*					

		about the future, since there is nothing that I can do about it anyway]					
**40**	**13**	Realizzo i miei progetti in tempo, facendo progressi regolari (F) [*I complete projects on time by making steady progress*]					
**42**	**14**	Faccio cose rischiose per rendere eccitante la mia vita (PH)					
**45**	**15**	Non ho difficoltà a resistere alle tentazioni quando so che c'è del lavoro da fare (F) [*I am able to resist temptation when I know that there is work to be done*]					
**50**	**16**	Penso alle cose brutte che mi sono accadute in passato (PN) [*I think about the bad things that have happened to me in the past*]					
